# A Multimodal Assessment of a New Self-Adhering Flowable Composite: Dental Pulp Stem Cell Viability and Bond Strength

**DOI:** 10.3390/jfb17070314

**Published:** 2026-06-29

**Authors:** Jiayuan Zhang, Chiharu Kawamoto, Ryotaro Yago, Hidehiko Sano, Fumiko Minowa, Asiful Islam, Rin Miyake, Yunqing Liu, Atsushi Tomokiyo

**Affiliations:** 1 Department of Restorative Dentistry, Faculty of Dental Medicine, Hokkaido University, Sapporo 0608586, Japan; zhangjiayuan@den.hokudai.ac.jp (J.Z.); chipa@den.hokudai.ac.jp (C.K.); ryago@den.hokudai.ac.jp (R.Y.); sano@den.hokudai.ac.jp (H.S.); minowa.fumiko.c5@elms.hokudai.ac.jp (F.M.); asiful5016@den.hokudai.ac.jp (A.I.); miyake.rin.g9@elms.hokudai.ac.jp (R.M.); 2 Department of Oral and Maxillofacial Surgery, The Second Affiliated Hospital of Harbin Medical University, Harbin 150086, China; 3 Department of Stomatology, The Forth Affiliated Hospital of Harbin Medical University, Harbin 150001, China; 4 Heilongjiang Provincial Key Laboratory of Hard Tissue Development and Regeneration, The Second Affiliated Hospital of Harbin Medical University, Harbin 150086, China

**Keywords:** self-adhering flowable composite, dentin bond strength, microhardness, biocompatibility, dental pulp stem cells

## Abstract

Self-adhering flowable composites (SAFCs) were developed to simplify restorative procedures by eliminating separate adhesive steps; however, their bonding performance, mechanical properties, and biological properties remain controversial. This study evaluated a newly developed SAFC (SA-100R) compared with three commercial SAFCs (Fusio Liquid Dentin, Constic, and Vertise Flow) and a conventional composite (Clearfil AP-X used with a two-step self-etch adhesive). Microtensile bond strength (μTBS) to dentin, failure modes, Knoop microhardness, resin–dentin interfacial morphology (SEM), elemental composition (EDX), and cytocompatibility (CCK-8, LIVE/DEAD staining) were assessed. The conventional composite system exhibited significantly higher µTBS than all SAFCs (*p* < 0.05). Among SAFCs, SA-100R showed the highest bond strength and no pre-testing failures. SEM revealed abundant resin tag formation only in the conventional system, while all SAFCs showed interfacial gaps, with SA-100R exhibiting the least pronounced gaps. SA-100R showed no significant cytotoxic effects across all tested concentrations. Within the limitations of this short-term in vitro investigation, SA-100R demonstrated improved bonding performance and favorable biocompatibility compared with existing SAFCs, suggesting its potential as a simplified restorative material. However, the conventional adhesive/composite system remains the reference standard for dentin bond strength, and long-term clinical performance remains to be established through further studies.

## 1. Introduction

Resin-based composite materials have become the primary choice for direct restorative procedures because of their favorable esthetics, adhesive capability, and minimally invasive application concepts [1]. The long-term clinical success of resin composite restorations, however, depends largely on the establishment of a stable and durable bond between the restorative material and dental hard tissues. Conventional adhesive procedures generally involve multiple clinical steps, including acid etching, priming, adhesive application, and incremental composite placement. Although these multi-step adhesive systems can achieve reliable dentin bonding and excellent clinical performance, they remain technique-sensitive and susceptible to operator-related variables such as moisture contamination, incomplete solvent evaporation, and inadequate adhesive infiltration [2,3]. In clinical situations involving limited operating time, poor isolation, pediatric patients, or difficult intraoral access, simplification of restorative procedures remains highly desirable. To address these limitations, self-adhering flowable composites (SAFCs) were developed as restorative materials capable of simultaneously providing adhesive and restorative functions within a single application step [2,4,5]. By incorporating acidic functional monomers into their resin matrix, SAFCs aim to eliminate the need for separate etching, priming, and bonding steps, thereby reducing technique sensitivity, minimizing contamination risks, and saving valuable chairside time [4,6,7].

Since the introduction of the first commercially available SAFCs-Fusio Liquid Dentin (Pentron, Orange, CA, USA) in 2009 and Vertise Flow (Kerr Corporation, Orange, CA, USA) in 2010-these materials have generated considerable research interest [8,9,10,11,12,13,14,15,16]. Despite their simplified clinical protocol, previous investigations have reported that the dentin bond strength of SAFCs remains generally inferior to that achieved by conventional adhesive/composite systems [17,18,19]. Unlike dedicated adhesive systems, SAFCs must simultaneously accomplish smear-layer modification, dentin infiltration, and restorative polymerization within a highly filled and relatively viscous material. These competing requirements may compromise the formation of an optimal hybrid layer and limit resin penetration into the dentin substrate. Studies have shown that self-adhering flowable composites (SAFCs) achieve better bonding strength and marginal sealing when used with a separate adhesive system than when used alone. This indicates that current SAFCs alone still fail to provide satisfactory adhesion to tooth hard tissues, and therefore have not yet achieved the goal of simplifying restorative procedures [20].

The adhesive behavior of SAFCs is strongly influenced by their monomer composition and filler characteristics. Functional acidic monomers such as 10-methacryloyloxydecyl dihydrogen phosphate (10-MDP), glycerol phosphate dimethacrylate (GPDM), and 4-methacryloxyethyl trimellitic acid (4-MET) have been incorporated into different formulations to promote chemical interaction with hydroxyapatite [21]. In addition, the balance between hydrophilic and hydrophobic resin components, filler loading, filler morphology, and polymer network structure may substantially influence the mechanical properties, polymerization behavior, water sorption, and biological performance of these materials.

Recently, a novel experimental SAFC designated as SA-100R (Kuraray Noritake Dental, Tokyo, Japan) has been developed with the aim of overcoming the limitations of existing materials. The formulation differs substantially from previously marketed SAFCs incorporating 10-MDP as the functional monomer and a hydrophilic amide monomer while avoiding HEMA/TEGDMA (Table 1), and may potentially enhance dentin interaction, polymer network stability, and mechanical performance. However, despite these potentially advantageous compositional characteristics, information regarding its dentin bonding efficacy, interfacial adaptation, mechanical behavior, and biocompatibility remains limited.

In addition to adhesive performance, the biological safety of resin-based restorative materials is an important clinical concern. Residual monomers such as triethylene glycol dimethacrylate (TEGDMA) and hydroxyethyl methacrylate (HEMA), released from incompletely polymerized resin composites may diffuse through dentinal tubules and induce cytotoxic, oxidative, or inflammatory responses in pulpal tissues [22,23,24,25]. Human dental pulp stem cells (hDPSCs) and mesenchymal stem cells (hMSCs) serve as relevant models for assessing such biological effects [26], given their role in pulpal defense and regenerative capacity. This is particularly clinically relevant when SAFCs are placed in deep cavities or pediatric restorations with limited remaining dentin thickness. The mechanical properties of restorative materials also play a crucial role in their long-term clinical performance. Surface microhardness is commonly used as an indirect indicator of polymerization efficiency, filler reinforcement, and resistance to surface deformation under occlusal loading. Furthermore, interfacial morphological analysis using scanning electron microscopy (SEM) may provide important information regarding hybridization quality, interfacial adaptation, and failure mechanisms at the resin–dentin interface [27]. Correlating these structural observations with bond strength and biological behavior may contribute to a more comprehensive understanding of the overall performance of SAFC materials.

Therefore, the aim of the present study was to comprehensively evaluate the dentin bonding performance, interfacial morphology, mechanical properties, and biocompatibility of the novel SAFC SA-100R in comparison with three commercially available SAFCs (Constic, Fusio Liquid Dentin, and Vertise Flow) and a conventional adhesive/composite system (Clearfil Megabond 2/Clearfil AP-X). Specifically, microtensile bond strength (μTBS), failure mode distribution, resin–dentin interfacial morphology, Knoop microhardness, and cytotoxic effects on hDPSCs and hMSCs were evaluated. The null hypothesis was that no significant differences would exist among the tested materials regarding these properties.

## 2. Materials and Methods

Human third molars extracted for orthodontic reasons were used in this study. A total of fifty-five extracted, sound human third molars were collected, preserved in periodically changed 0.5% chloramine-T solution at 4 °C for no longer than six months. This project was approved by the Ethics Committee of Hokkaido University Faculty of Dentistry (#2018-09). The materials used in this study are shown in Table 1, which includes four self-adhering flowable composites (SAFCs). Additionally, one conventional resin composite used with a two-step self-etch system was also examined in this experiment.

### 2.1. Experimental Design and Bonding Procedures

Flat mid-coronal dentin surfaces were exposed parallel to the occlusal surface using a Model Trimmer (MT-7; J. Morita, Tokyo, Japan) under continuous water cooling. The smear layers were standardized by polishing with 600-grit silicon carbide paper (Fuji Star Type DDC; Sankyo Rikagaku, Saitama, Japan). The prepared teeth were randomly assigned to five groups based on the materials applied. Four SAFCs (SA-100R, Fusio Liquid Dentin, Constic, and Vertise Flow) were applied to the dentin surfaces and incrementally built up to a height of 4 mm. In the combination group (Clearfil Megabond 2/Clearfil AP-X), an adhesive procedure was first performed, followed by composite build-up to 4 mm using a conventional resin composite. All bonding and restorative procedures were conducted strictly in accordance with the manufacturers’ instructions (Table 1). Light curing was performed using a light-curing unit (PenCure 2000; J Morita, Osaka, Japan) with an output intensity of 1000 mW/cm^2^. After restoration, all specimens were stored in distilled water at 37 °C for 24 h. To minimize operator variability, all procedures were performed by a single calibrated operator.

### 2.2. Microtensile Bond Strength (μTBS) Test

μTBS testing was conducted in accordance with the guidelines provided by the Academy of Dental Materials. Bonded teeth (*n* = 10 per group) were sectioned perpendicular to the bonding interface using a low-speed diamond saw (Isomet; Buhler, Lake Bluff, IL, USA) to obtain resin–dentin beams with a cross-sectional area of approximately 1 mm^2^. Each beam was attached to a Ciucchi’s jig using cyanoacrylate glue (Model Repair 2 Blue; J Morita). Tensile testing was conducted using a universal testing machine (EZ Test; Shimadzu; Kyoto, Japan) at a crosshead speed of 1 mm/min until failure. The μTBS value, expressed in megapascals (MPa), was calculated by dividing the failure force (in N) by the bonded area (in mm^2^). For statistical analysis, the mean μTBS value per tooth was considered as a single statistical unit.

### 2.3. Fracture Mode Analysis and SEM Observation

After the μTBS test, the fractured specimens were thoroughly dried overnight. They were then mounted onto aluminum stubs and sputter-coated with Pt-Pd alloy (E-1030, Hitachi, Tokyo, Japan) for 120 s. The specimens were observed using a scanning electron microscope (SEM, S-4000, Hitachi, Tokyo, Japan) at magnifications of 80× and 2000× with an accelerating voltage of 10 kV. The fracture modes were classified into adhesive failure (at the resin-dentin interface), cohesive failure in dentin, cohesive failure in resin composite, and mixed failure (combination of adhesive and cohesive).

### 2.4. Interfacial Structure Observation

For interfacial observation analysis, one representative tooth per group was examined. This sample size was chosen for qualitative morphological observation and is not intended for statistical comparison. The teeth were prepared and sectioned perpendicular to the bonding interface to obtain slabs with a thickness of 1.5 mm. The slab surfaces were sequentially polished using 600-, 800-, 1000-, and 1200-grit SiC papers under running water. The surfaces were then wet-polished sequentially with 6, 3, 1, and 0.25 μm diamond pastes (DP-Paste, Struers, Ballerup, Denmark). Specimens were ultrasonically cleaned for 5 min and treated with 1 M hydrochloric acid (HCl) for 10 s, followed by a 5.25 wt.% sodium hypochlorite (NaOCl) solution for 5 min to remove mineral and organic components, respectively. After dehydration for 24 h, specimens were sputter-coated with Pt-Pd and observed using SEM (S-4000; Hitachi) at 10 kV.

### 2.5. Knoop Microhardness Test

Five disc-shaped specimens per material were prepared at room temperature (23 ± 2 °C) and relative humidity of 50 ± 10%. Resin materials were dispensed directly into a Teflon mold (8 mm in diameter × 0.5 mm thick), covered with Mylar strips and glass slides. The material was carefully handled during the injection process to minimize entrapped air bubbles. The excess resin was removed by slight pressure. The resin was then light- cured for 40 s at 1000 mW/cm^2^. After curing, specimens were stored at 37 °C in a dark environment for 24 h. Surfaces were sequentially polished using SiC papers and diamond pastes down to 0.25 μm, followed by ultrasonic cleaning.

Knoop microhardness was measured using a microhardness tester (MVK-C; Akashi, Kanagawa, Japan). A load of 50 gf was applied with a dwell time of 15 s. For each sample, ten indentations were made radially from the center at intervals of 0.5 mm. The length of the long diagonal of each impression was measured, and the Knoop Hardness Number (KHN) was calculated according to the following formula:

$$KHN=\frac{P\;(g)}{7.028\times L_{2}\;(\mu^{2})}\times \;10^{5}$$

where *P* is applied load in grams (g), and *L* is the length of the long diagonal of the indentation in micrometers (*μ* = 0.001 mm). The mean microhardness value was determined from five samples per experimental group.

### 2.6. Elemental Analysis by Scanning Electron Microscope and Energy-Dispersive X-Ray Spectroscopy

Elemental composition of the resin materials was analyzed using SEM coupled with energy-dispersive X-ray spectroscopy (EDX) to descriptively characterize elements. Five resin discs per group were prepared and the resin surfaces were mechanically polished following the previously outlined procedures. The specimens were sputter-coated with silver for 60 s using a coating machine (JEOL Auto Fine Coater JFC-1600; JEOL Ltd., Akishima, Tokyo, Japan). Elemental analysis was performed using a SEM (JSM-6510LA; JEOL Ltd., Tokyo, Japan) equipped with a silicon drift detector (SDD). The measurement parameters were as follows: accelerating voltage 25.0 kV, irradiation current 1.00000 nA, energy range 0–20 keV, dwell time 0.50 ms, and number of sweeps 10. Qualitative analysis was used to identify elemental peaks, and semi-quantitative analysis was performed using standardless ZAF correction, with results expressed as weight% and atomic%. Due to the limitations of EDX in detecting light elements and the potential influence of coating and measurement conditions, the results were considered semi-quantitative and interpreted with caution.

### 2.7. Preparation of Material Extracts

Cylindrical specimens (5.0 ± 0.1 mm diameter and 2.0 ± 0.1 mm height) with a total surface area of approximately 0.71 cm^2^ were prepared with a Teflon mold for each material. The surface of each specimen was covered with a transparent Mylar strip and glass slides to prevent oxygen inhibition, avoid air bubbles, and ensure a flat surface [28,29]. Both sides of each sample were light-irradiated for 40 s at 1000 mW/cm^2^ to ensure adequate polymerization. After curing, the excess material was removed using 1000-grit SiC paper and stored at 37 °C and 100% humidity in a dark incubator for 24 h. The indirect contact method using material extracts was employed, as this approach is routinely used for cytotoxicity assessment of cured dental resin-based materials and allows standardized evaluation of leachable components. Samples were then immersed in Minimum Essential Medium Alpha Medium (α-MEM; Gibco Life Technologies Corporation, Grand Island, NE, USA) supplemented with 10% fetal bovine serum (FBS) and 1% penicillin/streptomycin (Gibco Life Technologies Corporation) at 37 °C in a humidified atmosphere of 5% CO_2_. Extraction was performed following ISO 10993-12 [30] with a surface area-to-volume ratio of 0.33 mL/cm^2^ [31]. After 3 days, eluates were collected and sterilized using a 0.2 μm filter.

### 2.8. Cell Culture and Cytotoxicity Assay

Human dental pulp stem cells (hDPSCs; Catalog #: PT-5025, Lonza Biologics, Slough, UK) were purchased from Lonza Biologics. Primary human umbilical cord mesenchymal stem cells lines (hMSCs) were kindly provided by Heilongjiang provincial key laboratory of hard tissue development and regeneration, the second affiliated hospital of Harbin Medical University. Both cell types were cultured in α-MEM supplemented with 10% FBS, and 1% penicillin/streptomycin at 37 °C in a humidified atmosphere of 5% CO_2_. The culture medium was changed every 2–3 days, and cells were passaged when they reached 80% confluence.

For cytotoxicity testing, a density of 1.0 × 10^4^ cells per well was seeded in 96-well plates. After 24 h, they were exposed to extracts (200 μL/well) at serial dilutions (1:1 to 1:16) with cell culture medium for another 2 days. The cells treated with complete media represent the negative control group. Each condition was assessed in triplicate in three independent assays. Cell viability was assessed using a Cell Counting Kit-8 (CCK-8; Dojindo, Kumamoto, Japan) according to manufacturer’s protocol. Absorbance at 450 nm was then measured using a microplate reader (Bio-Rad Laboratories, Hercules, CA, USA). The cell viability was standardized to the relative control as a percentage.

### 2.9. LIVE and DEAD Staining

To assess the reliability of the assay, the cell viability of hDPSCs and hMSCs exposed to serial dilutions of extracts was evaluated using the LIVE/DEAD assay. Briefly, cells were seeded in the 8-chamber slides (Lab-Tek^®^; ThermoFisher Scientific, Waltham, MA, USA) at a density of 1.0 × 10^4^ cells/well and incubated for 24 h to allow for cell adhesion, as described previously. After the adhesion period, the culture medium in each well was replaced with 200 μL of serial dilutions of the test materials, and cells were incubated for an additional 24 h. Following incubation, the cells were washed three times with PBS. According to the manufacturer’s instructions, the cells were then stained using the LIVE/DEAD Cell Imaging Kit (488/570) (Invitrogen, Waltham, MA, USA). Fluorescent images were captured using an Olympus FV300 fluorescent microscope (Olympus Corporation, Tokyo, Japan) at 10× magnification.

### 2.10. Statistical Analysis

The sample size was calculated using G*Power software (version 3.1.9.7, Heinrich-Heine University, Düsseldorf, Germany). Based on a pilot study (*n* = 5 teeth per group), a very large effect size (Cohen’s f = 3.59) was observed for μTBS among the five material groups. Applying an alpha level of 0.05 and a statistical power of 0.80, the analysis resulted in a minimum required sample size of 2–3 teeth per group. Nevertheless, to account for technical variability and ensure robust variance estimation, a conservative sample size of 10 measurable teeth per group was used. This sample size is consistent with typical μTBS studies (5–12 teeth per group) [32,33,34]. The sample size for cell viability assays (three independent experiments with triplicate wells) followed common practice in cytotoxicity studies [28]. Statistical analyses were performed using GraphPad Prism 10.0 (GraphPad Software Inc., San Diego, CA, USA). Data are presented as mean ± standard deviation. Normality of all quantitative datasets was assessed using the Shapiro–Wilk test. Homogeneity of variances was examined using both Brown–Forsythe test and Bartlett’s test. For μTBS, variances were heterogeneous (*p* < 0.0001); therefore, Welch’s ANOVA and Brown–Forsythe ANOVA were applied, followed by Dunnett’s T3 multiple comparisons test. For KHN and CCK-8, homogeneity of variances was confirmed; thus, one-way ANOVA was applied (Tukey’s post hoc for KHN, and Dunnett’s post hoc for CCK-8). LIVE/DEAD staining was evaluated qualitatively, and no statistical analysis was applied. Statistical significance was set at *p* < 0.05.

## 3. Results

### 3.1. Microtensile Bond Strength Test and Failure Modes Analysis

**Figure 1:**
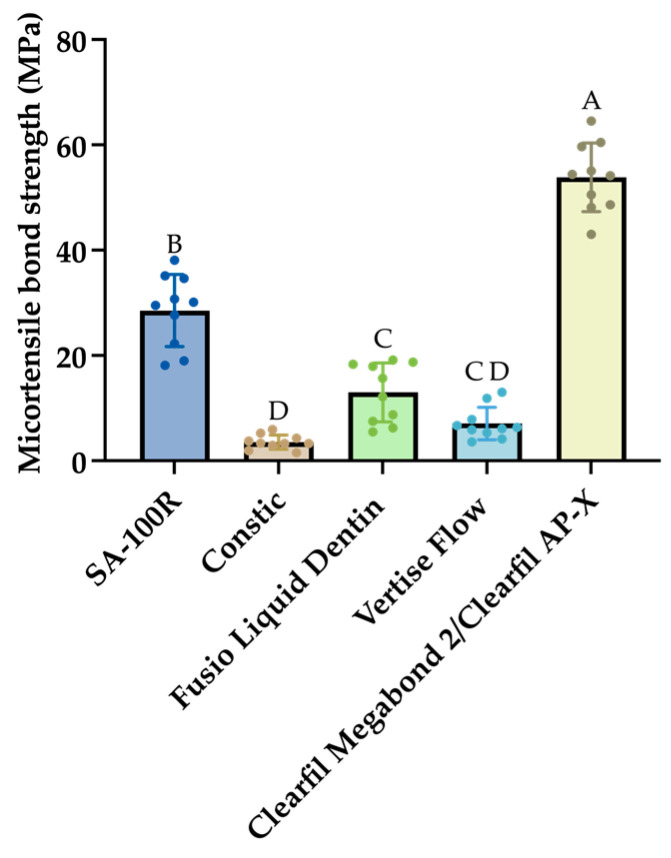
μTBS results of dentin–resin composite samples after 24 h of water storage. Mean μTBS values (MPa) with standard deviation. Different letters indicate statistically significant differences among the groups (*p* < 0.05).

The μTBS values for all groups are presented in Figure 1 as a bar graph, displaying the mean ± SD values (*n* = 10). Pre-testing failure, referring to specimens that debonded prior to testing, and such specimens were assigned a μTBS value of 0 MPa. A one-way ANOVA statistical analysis revealed a significant influence on the μTBS values by the materials (F = 160.1, *p* < 0.0001). Overall, the Clearfil Megabond 2/Clearfil AP-X group demonstrated the highest μTBS value (53.85 ± 6.51 MPa). Among the self-adhesive materials, the value of SA-100R (28.53 ± 6.84 MPa) was significantly higher than that of other materials. However, its μTBS remained substantially lower than that achieved by the conventional Clearfil Megabond 2/Clearfil AP-X system, which demonstrated approximately two-fold higher bond strength. In addition, there was no significant difference in μTBS values between Fusio Liquid Dentin (12.99 ± 5.59 MPa) and Vertise Flow (7.090 ± 3.09 MPa), while the Constic group showed the lowest values (3.551 ± 1.35 MPa).

### 3.2. Failure Modes Analysis and SEM Observation

**Figure 2:**
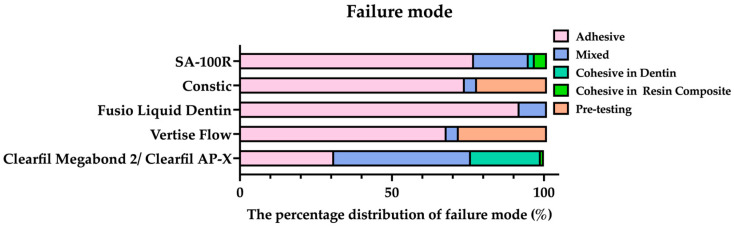
Bar graphs represent the percentage distribution of failure mode within each group, including the pre-testing failures.

The percentage distribution of failure modes detected in this study is shown in Figure 2 and the failure mode varied among the different materials tested. Among the self-adhering flowable composites, adhesive failure predominated in all groups, with Fusio Liquid Dentin showing the highest rate (91.5%), followed by SA-100R (76.6%), Constic (73.9%), and Vertise Flow (67.8%). Notably, pre-testing failures occurred only in Constic (22.6%) and Vertise Flow (28.7%), correlating with their low μTBS values.

**Figure 3:**
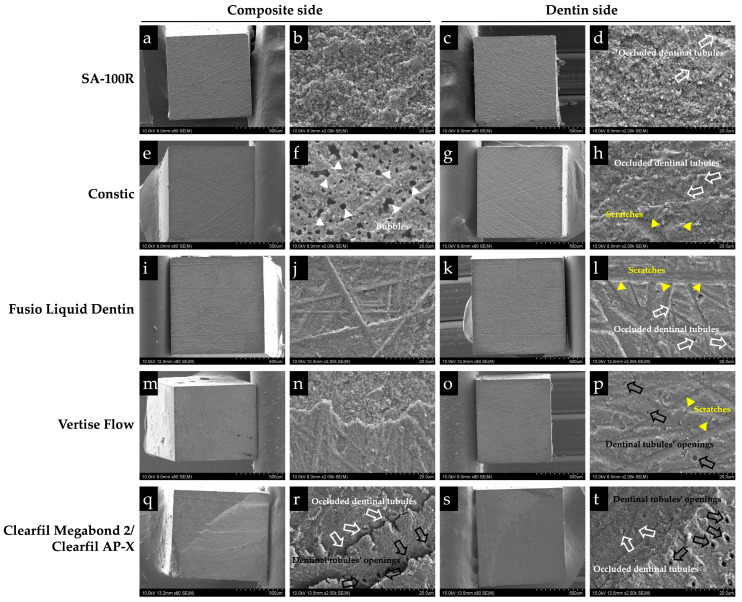
Representative SEM images of the fractured resin composite and dentin surface of each group’s after 24 h at ×80 (**a**,**c**,**e**,**g**,**i**,**k**,**m**,**o**,**q**,**s**) and ×2000 (**b**,**d**,**f**,**h**,**j**,**l**,**n**,**p**,**r**,**t**) magnifications. The black arrows indicate the dentinal tubules’ openings, and the white arrows depict partially occluded dentinal tubules. The yellow arrowheads indicate notable polishing scratches, and the white arrows indicate bubbles.

Representative SEM images of the adhesive failures are shown in all self-adhering flowable composite groups (Figure 3). On the corresponding dentin sides of Constic, Fusio Liquid Dentin and Vertise Flow groups, polishing scratches were clearly visible. Dentin tubules were observed to be plugged with resin tag in Constic and Fusio Liquid Dentin, while noticeable openings of dentinal tubules were observed in Vertise Flow (Figure 3h,l,p). In addition, numerous void structures were observed on the composite side of Constic (Figure 3f). In the conventional composite group (Clearfil Megabond 2/Clearfil AP-X), mixed failures (44.5%) and adhesive failures (30.8%) were frequently observed. Exposed dentin tubules could be detected on both the dentin and resin composite surface in Clearfil Megabond 2/Clearfil AP-X group, indicating mixed failure (Figure 3q–t).

### 3.3. Interfacial Morphology Observation

**Figure 4:**
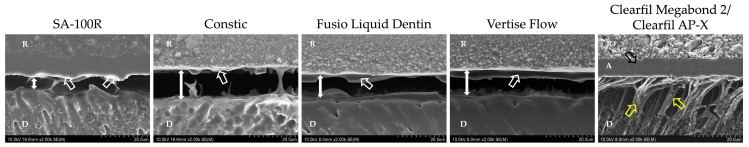
Representative SEM images of the resin–dentin interfaces of each group after 24 h (2000×). SA-100R exhibited minimal interfacial gaps, whereas Constic, Fusio Liquid Dentin, and Vertise Flow showed more pronounced interfacial separation. In the Clearfil Megabond 2/Clearfil AP-X group, abundant resin tags were observed. White double-headed arrows indicate interfacial gaps; white arrows indicate the hybrid layer; black arrows indicate the adhesive layer; yellow arrows indicate resin tags. R, resin; D, dentin; A, adhesive.

Figure 4 presents representative SEM images of the resin–dentin interfaces. In the Clearfil Megabond 2/Clearfil AP-X group, numerous long and densely distributed resin tags were clearly observed, indicating effective micromechanical interlocking. In contrast, all SARCs demonstrated interfacial debonding to varying extents. Among them, SA-100R exhibited the smallest and least continuous gaps, whereas markedly wider and more distinct interfacial separations were observed in Constic, Fusio Liquid Dentin, and Vertise Flow.

### 3.4. Knoop Hardness Test

**Figure 5:**
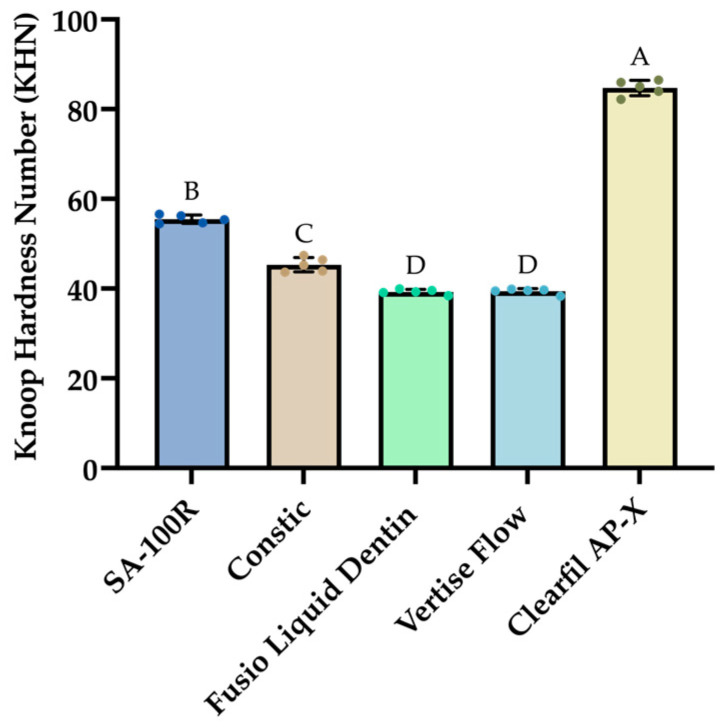
Bar graphs showing mean KHN values (standard deviation) of the materials. Different letters indicate statistically significant differences among the materials (*p* < 0.05).

The KHN results are presented in Figure 5. One-way ANOVA revealed that the material type had a significant effect on microhardness (*p* < 0.0001). Among all materials, Clearfil AP-X exhibited the highest microhardness value (84.71 ± 1.712). Among the SAFC materials, SA-100R showed the greatest hardness (55.45 ± 0.9394), which was significantly higher than Constic, Fusio Liquid Dentin, and Vertise Flow. No significant difference was observed between Fusio Liquid Dentin and Vertise Flow (*p* > 0.05).

### 3.5. Scanning Electron Microscope and Energy-Dispersive X-Ray Spectroscopy Examination

**Figure 6:**
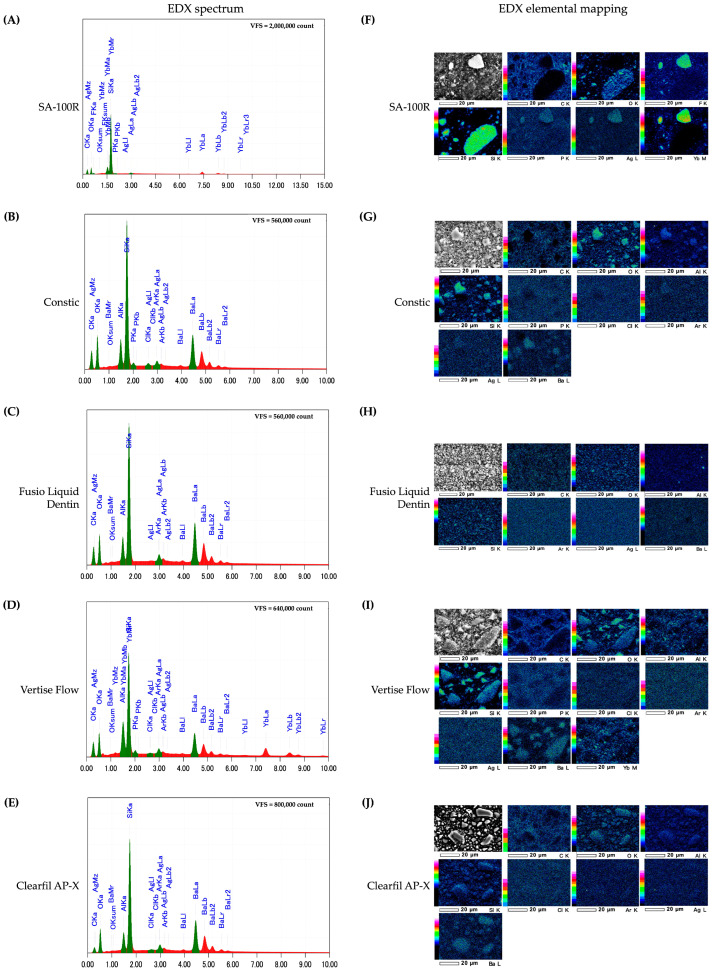
EDX analysis of SA-100R, Constic, Fusio Liquid Dentin, Vertise Flow, and Clearfil AP-X. (**A**–**E**) EDX spectral analysis and elemental composition of each material. (**F**–**J**) SEM/EDX elemental distribution maps (×1000 magnification) of each material.

The elemental composition of each resin composite was evaluated by SEM/EDX (Figure 6). Carbon (C), oxygen (O), and silicon (Si) were the predominant elements detected in all materials, consistent with the organic matrix and inorganic fillers. Additional elements such as aluminum (Al), barium (Ba), and ytterbium (Yb) were detected in some materials, reflecting differences in filler composition. Given the semi-quantitative nature of EDX and methodological limitations, these results should be interpreted as indicative rather than absolute.

### 3.6. In Vitro Cytotoxicity Analysis

**Figure 7:**
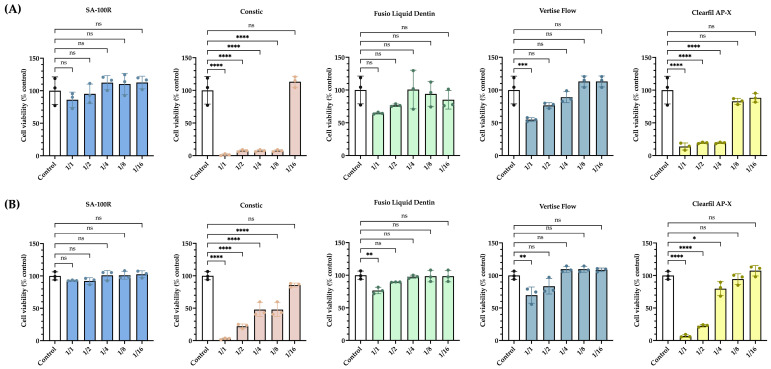
Cell viability was assessed using the CCK8 assay to evaluate the effects of SA-100R, Constic, Fusio Liquid Dentin, Vertise Flow, and Clearfil AP-X at different dilution concentrations. (**A**) shows the cell viability of hDPSCs. (**B**) shows the cell viability of hMSCs. Asterisks indicate a significant difference from the Control group (Kruskal–Wallis test, * *p* < 0.05, ** *p* < 0.01, *** *p* < 0.001, **** *p* < 0.0001).

The results of in vitro cytotoxicity against hDPSCs and hMSCs for the serial dilutions of extracts of materials are shown in Figure 7. As shown in Figure 7A, compared to the untreated control group, the two materials—Constic and Clearfil AP-X—significantly reduced the metabolic activity of hDPSCs when used in undiluted form (1/1). Notably, Constic, when diluted to a 1/16 concentration (*p* < 0.0001), and Clearfil AP-X, when diluted to a 1/8 concentration (*p* < 0.0001), did not exhibit any noticeable effects on the cell viability of hDPSCs. Vertise Flow, in its undiluted state (1/1), also caused a significant reduction in the metabolic activity of hDPSCs. In contrast, SA-100R and Fusio Liquid Dentin showed no significant effect on the growth of hDPSCs across all dilution concentrations (*p* > 0.05).

In Figure 7B, Constic and Clearfil AP-X exhibited clear cytotoxicity to hMSCs at higher dilution concentrations. Vertise Flow and Fusio Liquid Dentin caused a significant decrease in the metabolic activity of hDPSCs when used undiluted (1/1). SA-100R, however, had no apparent impact on the growth of hMSCs at any dilution concentration (*p* > 0.05). These results suggest that SA-100R exhibits the best biocompatibility among the five tested materials.

### 3.7. LIVE and DEAD Observation

**Figure 8:**
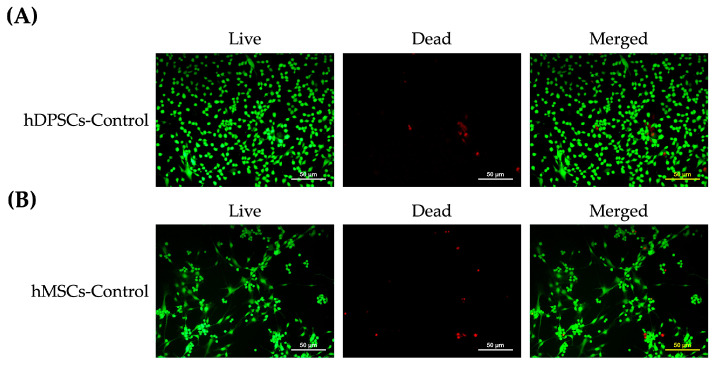
LIVE/DEAD staining for Control group in (**A**) hDPSCs and (**B**) hMSCs for 48 h. Green indicates live cells, and red indicates dead cells. Scale bar = 50 μm.

To assess cell viability and the distribution of live and dead cells after 48 h of incubation, the LIVE/DEAD assay was performed on two different cell lines, hDPSCs and hMSCs. The results presented in Figure 8A,B represent the control group, where untreated cells in both hDPSCs (Figure 8A) and hMSCs (Figure 8B) exhibit intact and well-defined nuclei, appearing green, with only a few cells displaying red fluorescence, indicating dead cells.

**Figure 9:**
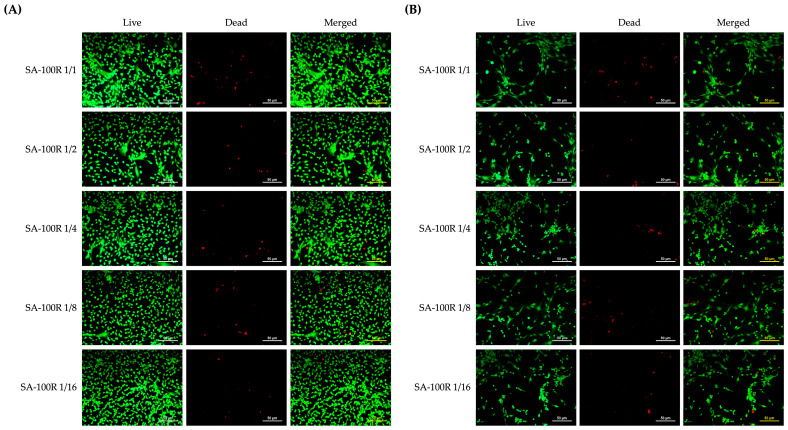
LIVE/DEAD staining of hDPSCs and hMSCs treated with serial dilutions of SA-100R. (**A**) Representative LIVE/DEAD images of hDPSCs exposed to serial dilutions of SA-100R for 48 h. (**B**) Representative LIVE/DEAD images of hMSCs exposed to serial dilutions of SA-100R for 48 h. Green indicates live cells, and red indicates dead cells. Scale bar = 50 μm.

Figure 9A and Figure 9B illustrate the effects of serial dilutions of SA-100R extracts on hDPSCs and hMSCs, respectively. The images demonstrate that at all tested concentrations, both cell types exhibited strong green fluorescence, indicating live cells, with minimal red fluorescence, suggesting a small number of dead cells. The intensity of green and red fluorescence appeared similar across the different concentrations of SA-100R and comparable to that of the control group. These findings suggest that SA-100R does not induce toxicity in either hDPSCs or hMSCs, corroborating the results from the CCK-8 assay and indicating good biocompatibility.

**Figure 10:**
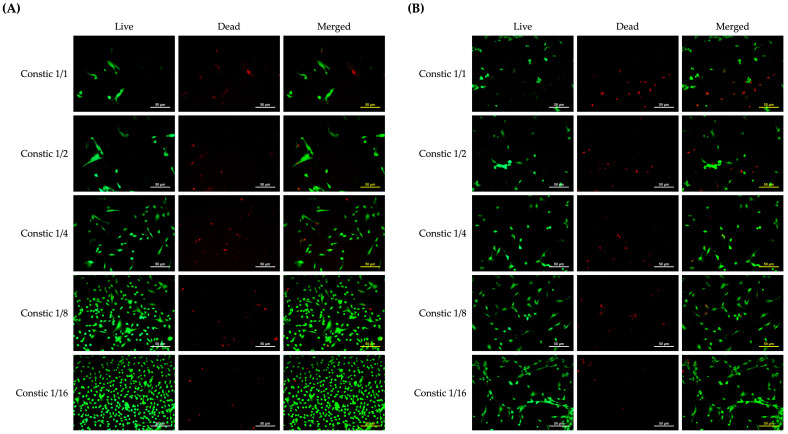
LIVE/DEAD staining of hDPSCs and hMSCs treated with serial dilutions of Constic. (**A**) Representative LIVE/DEAD images of hDPSCs exposed to serial dilutions of Constic for 48 h. (**B**) Representative LIVE/DEAD images of hMSCs exposed to serial dilutions of Constic for 48 h. Green indicates live cells, and red indicates dead cells. Scale bar = 50 μm.

When comparing Constic 1/1 (the undiluted extract concentration) to the control group, a reduction in live cells (green fluorescence) and an increase in dead cells (red fluorescence) were observed in both hDPSCs (Figure 10A) and hMSCs (Figure 10B). In hDPSCs, as the concentration of Constic decreased (from 1/1 to 1/16), the number of live cells increased, while the amount of dead cells decreased. A similar trend was observed in hMSCs, where lower concentrations of Constic led to a higher proportion of live cells and a reduction in dead cells.

**Figure 11:**
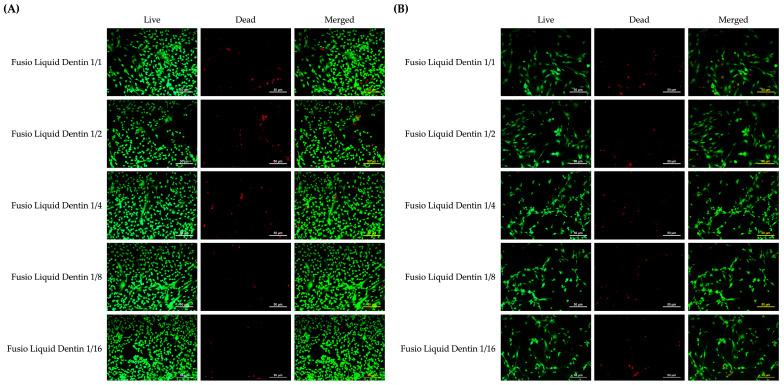
LIVE/DEAD staining of hDPSCs and hMSCs treated with serial dilutions of Fusio Liquid Dentin. (**A**) Representative LIVE/DEAD images of hDPSCs exposed to serial dilutions of Fusio Liquid Dentin for 48 h. (**B**) Representative LIVE/DEAD images of hMSCs exposed to serial dilutions of Fusio Liquid Dentin for 48 h. Green indicates live cells, and red indicates dead cells. Scale bar = 50 μm.

Figure 11 shows the LIVE/DEAD images for Fusio Liquid Dentin. In Figure 11A (hDPSCs), no differences in the proportions of live and dead cells were observed among any of the extract concentration groups or between any group and the control. In Figure 11B (hMSCs), only the high concentration of Fusio Liquid Dentin (1/1) resulted in a slight increase in dead cells compared to the control and other dilution groups, while the number of live cells remained similar to the control. For concentrations ranging from 1/16 to 1/2, the proportions of live and dead cells were comparable, with no differences observed.

**Figure 12:**
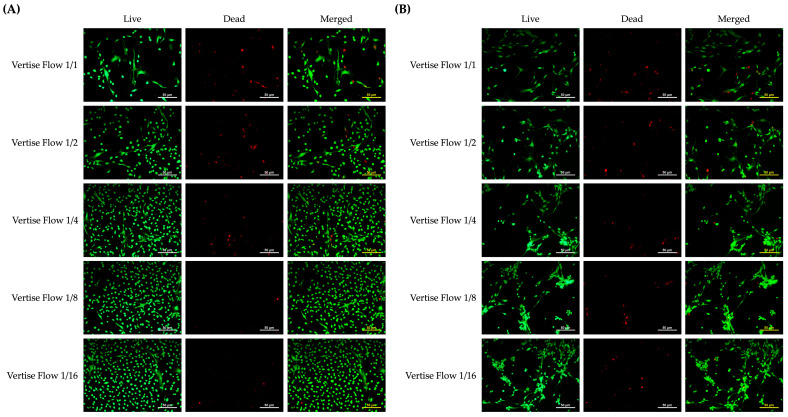
LIVE/DEAD staining of hDPSCs and hMSCs treated with serial dilutions of Vertise Flow. (**A**) Representative LIVE/DEAD images of hDPSCs exposed to serial dilutions of Vertise Flow for 48 h. (**B**) Representative LIVE/DEAD images of hMSCs exposed to serial dilutions of Vertise Flow for 48 h. Green indicates live cells, and red indicates dead cells. Scale bar = 50 μm.

Figure 12A shows that Vertise Flow at concentrations of 1/1 and 1/2 affected hDPSCs, leading to a decrease in live cells and a slight increase in dead cells compared to the control group. As the concentration decreased, the number of live cells increased, showing no difference from the control group, and no differences were observed between the groups. In hMSCs (Figure 12B), Vertise Flow at concentrations of 1/1, 1/2, and 1/4 also resulted in a reduction in live cells, while at concentrations of 1/8 and 1/16, the proportions of live and dead cells were similar to those in the control group.

**Figure 13:**
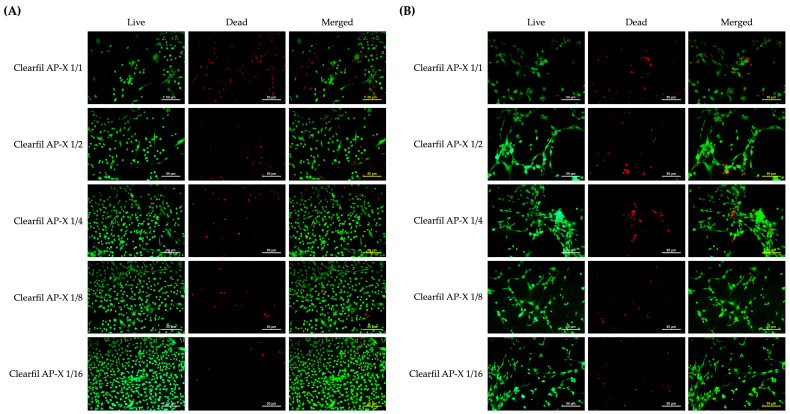
LIVE/DEAD staining of hDPSCs and hMSCs treated with serial dilutions of Clearfil AP-X. (**A**) Representative LIVE/DEAD images of hDPSCs exposed to serial dilutions of Clearfil AP-X for 48 h. (**B**) Representative LIVE/DEAD images of hMSCs exposed to serial dilutions of Clearfil AP-X for 48 h. Green indicates live cells, and red indicates dead cells. Scale bar = 50 μm.

Compared to the control group, the undiluted Clearfil AP-X (1/1 concentration) caused a decrease in live cells in hDPSCs, accompanied by a marked increase in dead cells (Figure 13A). At concentrations of 1/2 and 1/4, live cell numbers were also reduced, but remained higher than at the 1/1 concentration, with a corresponding decrease in dead cells (red fluorescence) compared to the 1/1 group. No differences in the numbers of live or dead cells were observed between the 1/8 and 1/16 groups and the control group. In hMSCs (Figure 13B), the undiluted Clearfil AP-X (1/1 concentration) led to a reduction in live cells, with fewer live cells than in the control and other concentration groups. At concentrations of 1/2 and 1/4, live cell numbers were similar to those in the control group, while dead cells (red fluorescence) were increased compared to the control group. No differences in live or dead cells were observed at the 1/8 and 1/16 concentrations when compared to the control group.

## 4. Discussion

The present study comprehensively evaluated the dentin bonding performance, interfacial morphology, mechanical properties, and biocompatibility of a newly developed self-adhering flowable composite (SA-100R) in comparison with currently available self-adhering flowable composites (SAFCs) and a conventional adhesive/composite system. Overall, the null hypothesis was partially rejected because significant differences were observed among the tested materials in terms of μTBS, interfacial adaptation, hardness, and cytocompatibility. Among the SAFCs, SA-100R demonstrated the most favorable overall performance, particularly with respect to dentin bond strength and biological behavior.

Bond strength to dentin remains one of the most critical determinants of the long-term clinical success of resin restorations. Inadequate adhesion can result in marginal gap formation, nanoleakage, bacterial penetration, postoperative sensitivity, and secondary caries, ultimately compromising restoration longevity [35,36,37]. In the present study, the conventional composite system (Clearfil Megabond 2/Clearfil AP-X) exhibited significantly higher μTBS values than all SAFCs (Figure 1). This result is consistent with previous studies demonstrating that multi-step adhesive systems generally achieve superior and more durable dentin bonding compared with simplified self-adhering restorative materials [4,38]. The superior performance of Clearfil Megabond 2 can be attributed primarily to its well-established two-step self-etch bonding strategy based on 10-MDP functional monomer chemistry. The primer effectively infiltrates partially demineralized dentin, while the subsequent hydrophobic bonding resin promotes formation of a relatively uniform hybrid layer and stable micromechanical interlocking. In addition, 10-MDP is capable of forming stable calcium salts with residual hydroxyapatite, contributing to durable chemical adhesion at the dentin interface [39,40,41,42]. These mechanisms are reflected in the SEM observations, where numerous long and densely distributed resin tags and a distinct adhesive layer were observed in the Clearfil Megabond 2/Clearfil AP-X group (Figure 4).

In contrast, all SAFCs exhibited significantly lower μTBS values and predominantly adhesive failure patterns. Unlike conventional adhesive systems, SAFCs are required to simultaneously perform demineralization, infiltration, wetting, and polymerization within a single highly filled restorative material. This intrinsic design limitation reduces the ability of acidic functional monomers to adequately penetrate the smear layer and underlying dentin substrate. Consequently, formation of a homogeneous hybrid layer becomes difficult, leading to weak interfacial integrity. This interpretation is strongly supported by the interfacial SEM findings in the present study, in which all SAFCs exhibited varying degrees of interfacial debonding and gap formation (Figure 4). Moreover, adhesive failure predominated in all SAFC groups, indicating that the resin–dentin interface remained the weakest region within the bonded assembly.

Among the tested SAFCs, SA-100R demonstrated significantly higher μTBS values than Fusio Liquid Dentin, Vertise Flow, and Constic (Figure 1). Importantly, no pre-testing failures were observed in the SA-100R group, whereas substantial premature failures occurred in Constic and Vertise Flow (Figure 2). These findings suggest that the adhesive interface created by SA-100R was more stable and mechanically resistant than those produced by earlier-generation SAFCs. The improved bonding performance of SA-100R is likely associated with its optimized monomer system and resin formulation. First, SA-100R contains 10-MDP, which is regarded as one of the most effective functional monomers for dentin adhesion because of its ability to establish stable ionic bonding with hydroxyapatite. In contrast, Vertise Flow relies primarily on GPDM as its acidic monomer. Previous studies have suggested that GPDM-treated dentin is more hydrophilic than 10-MDP-treated one and that GPDM-Ca salts are more susceptible to hydrolytic degradation [43]. These factors likely explain the relatively poor bonding performance and high pre-testing failure rate observed for Vertise Flow. Fusio Liquid Dentin utilizes 4-MET as its functional adhesive monomer. Although 4-MET can interact chemically with dentin calcium, its bonding capability is generally considered weaker than that of 10-MDP-based systems. Furthermore, Fusio Liquid Dentin contains relatively hydrophilic monomers such as HEMA and TEGDMA, which may increase water sorption and compromise polymer network stability after polymerization [44]. The predominance of adhesive failure and the presence of interfacial gaps observed in Fusio Liquid Dentin are consistent with these characteristics. Interestingly, despite containing 10-MDP, Constic demonstrated the lowest μTBS values among all materials tested. This finding suggests that the presence of 10-MDP alone is insufficient to guarantee effective dentin bonding in SAFC systems. Constic contains a relatively complex resin matrix composed of Bis-GMA, UDMA, EBADMA, HEMA, TEGDMA, and HDMA. While these monomers may improve handling and flowability, excessive hydrophilic monomer content may negatively affect polymerization behavior and increase water sorption [45]. Moreover, numerous void structures were observed on the fractured composite surface of Constic, indicating possible incomplete adaptation or air entrapment during application. Such structural defects may act as stress concentration sites and further weaken interfacial integrity. The high percentage of pre-testing failures observed in Constic strongly supports this interpretation.

From a clinical perspective, a relevant question is whether the μTBS value of SA-100R (28.53 ± 6.84 MPa) is clinically acceptable despite being approximately half that of the conventional adhesive system. Although no universally accepted threshold exists, values exceeding 20–25 MPa are generally considered sufficient for small-to-moderate restorations with favorable cavity configurations (e.g., Class I, III, V). The bond strength of SA-100R compares favorably with other SAFCs reported in the literature (typically 5–20 MPa) [4,13,20,32,46,47]. Clinical evidence further supports this interpretation: SAFCs have demonstrated acceptable clinical performance in small Class I restorations and as pit and fissure sealants [48,49]. Therefore, SA-100R may be suitable for clinical situations where procedural simplification is a priority, such as small cervical lesions, pediatric restorations, or non-stress-bearing Class I cavities, whereas conventional multi-step adhesives remain the standard of care for large stress-bearing restorations.

Another important factor that may explain the superior bonding behavior of SA-100R is the possible contribution of its hydrophilic amide monomer. Amide-containing adhesive monomers have recently attracted attention because of their improved hydrolytic stability and enhanced hydrogen-bonding capability compared with conventional methacrylate systems. Such monomers may improve dentin wettability and facilitate more stable interfacial interaction under moist conditions [50,51]. In addition, SA-100R contains both hydrophilic and hydrophobic dimethacrylates, which may provide a more balanced resin matrix capable of simultaneously achieving dentin interaction and polymer network stability. This optimized monomer balance may explain why SA-100R exhibited smaller and less continuous interfacial gaps than the other SAFCs.

The interfacial morphology findings correlated closely with the μTBS results and failure mode analysis. In the Clearfil Megabond 2/Clearfil AP-X group, abundant resin tags and intimate adaptation to dentin were clearly observed, reflecting effective infiltration into dentinal tubules and formation of strong micromechanical retention. Conversely, all SAFCs demonstrated interfacial separation to varying extents, confirming their limited ability to establish stable hybridized interfaces. Among them, SA-100R showed the narrowest and least continuous gaps, whereas Constic, Fusio Liquid Dentin, and Vertise Flow exhibited more pronounced debonding. These observations support the concept that interfacial adaptation is strongly associated with bonding effectiveness.

The predominance of adhesive failures observed in all the SAFC groups in the present study further supports this interpretation (Figure 2), indicating that the adhesive interface remains the weakest link within the bonded assembly. Similar failure patterns have been reported in previous studies evaluating self-adhering composites, where incomplete interaction between functional monomers and the dentin substrate was identified as a primary factor limiting bonding performance [47]. By comparison, the conventional adhesive system exhibited a substantial proportion of mixed failures, suggesting that the interfacial bond strength approached or exceeded the cohesive strength of adjacent substrates. The exposed dentinal tubules observed on both dentin and composite surfaces in the conventional group are characteristic of mixed failure and further indicate stronger interfacial integrity (Figure 3).

Surface hardness analysis revealed significant differences among the tested materials. Clearfil AP-X exhibited the highest Knoop hardness values, which is consistent with its high filler loading (71 vol.%) and highly filled conventional composite formulation. Increased inorganic filler content may generally improve resistance to indentation and enhances overall mechanical performance [52,53]. By comparison, flowable composites typically contain lower filler fractions to maintain reduced viscosity and improved handling characteristics, which may compromise hardness. SA-100R demonstrated significantly greater hardness than Constic, Fusio Liquid Dentin, and Vertise Flow. This finding may be considered noteworthy, as SAFCs are often thought to have inferior mechanical properties-potentially resulting from their simpler formulations and lower filler content. Although the exact filler loading of SA-100R was not disclosed by the manufacturer, its relatively high hardness may be related to differences in filler composition and resin matrix formulation compared with the other self-adhering materials. In addition, the presence of hydrophobic aromatic dimethacrylate may contribute to increased rigidity of the cured polymer network [54]. Fusio Liquid Dentin and Vertise Flow exhibited the lowest hardness values among the tested materials, with no significant difference observed between them. Both materials contain lower filler fractions and relatively flowable resin matrices designed to facilitate adaptation to tooth substrates. Such formulations generally require increased resin content, which may reduce surface hardness because the softer organic matrix becomes more susceptible to localized deformation during indentation testing [52,55]. Moreover, the presence of pre-polymerized fillers in Vertise Flow may also influence load transfer efficiency within the material [56,57]. Importantly, the hardness results did not directly correlate with dentin bond strength. Although SA-100R demonstrated superior μTBS among the SAFCs, its hardness remained substantially lower than that of the conventional Clearfil Megabond 2/Clearfil AP-X system. This finding suggests that bonding effectiveness and surface hardness reflect different aspects of material performance. While interfacial bonding is influenced by interactions occurring at the resin–dentin interface, hardness primarily reflects the resistance of the cured material surface to localized deformation. Furthermore, Knoop hardness represents only one aspect of mechanical performance and cannot directly predict clinical wear resistance, fracture behavior, or long-term durability. Additional studies incorporating aging and wear evaluation are therefore required to determine the clinical significance of the observed hardness differences.

Elemental composition analysis using SEM/EDX further revealed distinct compositional differences among the materials. Carbon, oxygen, and silicon were identified as the predominant elements in all composites, reflecting the typical composition of organic resin matrices and silica-based fillers (Figure 6). Notably, fluorine was detected exclusively in SA-100R, suggesting the incorporation of fluoride-containing components in its formulation. Fluoride release from restorative materials has been associated with potential anticariogenic effects, including inhibition of enamel demineralization and promotion of remineralization processes in adjacent tooth structures [7]. The detection of fluorine exclusively in SA-100R may reflect differences in its fluoride-containing formulation. However, the present SEM/EDX data are not sufficient to establish a direct contribution of fluorine to the higher bond strength observed for SA-100R, and this possible relationship should be investigated in future studies. In addition, ytterbium detected in SA-100R and Vertise Flow likely serves as a radiopacifying agent, enhancing radiographic detection of restorations [55].

Biocompatibility is another essential consideration for resin-based restorative materials, particularly when placed in close proximity to dentin and pulp tissues [26,58]. According to ISO 10993-5 standards [31], a material is considered cytotoxic when cell viability decreases below 70% relative to negative controls. Residual monomers such as TEGDMA and HEMA may be released from incompletely polymerized composites and diffuse through dentinal tubules, potentially inducing cytotoxic or inflammatory responses in pulpal cells [25]. In the present study, cytotoxicity was evaluated using hDPSCs and hMSCs, two biologically relevant cell models for assessing pulp-related cellular responses. Remarkably, Constic and Clearfil AP-X demonstrated more pronounced cytotoxicity, while Vertise Flow and Fusio Liquid Dentin showed moderate concentration-dependent effects.

The observed cytotoxicity differences are likely associated with differences in monomer composition and elution behavior. Previous studies have demonstrated that common resin monomers such as TEGDMA, HEMA, and Bis-GMA can induce oxidative stress, apoptosis, inflammatory responses, and impaired differentiation in dental pulp cells. TEGDMA is a monomer known to exhibit greater cytotoxicity than other common resin components and to readily dissolve in saliva, which increases its potential for elution and subsequent biological interactions [8]. The consistently higher toxicity observed even at diluted concentrations suggests that Constic and Clearfil AP-X may contain higher proportions of TEGDMA or other cytotoxic monomers within their formulations. Vertise Flow and Fusio Liquid Dentin showed moderate cytotoxic effects in their undiluted forms. The observed concentration-dependent cytotoxicity suggests that dilution of leachable components may reduce their biological impact, which may partially explain why such materials remain clinically acceptable despite detectable in vitro cytotoxicity. In contrast, SA-100R exhibited the most favorable cytocompatibility profile among the tested materials, showing no significant reduction in the viability of hDPSCs or hMSCs across all dilution concentrations (Figure 7). This may be associated with the absence of HEMA and TEGDMA from its disclosed formulation. Instead, SA-100R contains urethane acrylate, hydrophobic dimethacrylates, and a hydrophilic amide monomer. Amide-containing monomers have recently attracted attention because they may exhibit lower water sorption and improved hydrolytic stability compared with conventional hydrophilic methacrylates such as HEMA. Moreover, the relatively hydrophobic nature of several SA-100R matrix components may reduce monomer elution after polymerization, thereby limiting cellular exposure to unreacted substances. These characteristics may partially explain why SA-100R maintained high cell viability even under undiluted extract conditions.

From a clinical perspective, the development of SAFCs aims to simplify restorative procedures by eliminating separate adhesive steps and reducing technique sensitivity. Such materials may be particularly advantageous in minimally invasive treatments, pediatric dentistry, or clinical situations requiring rapid restorative procedures [4,9,13,44,59]. The present findings contribute to the growing body of evidence evaluating the performance of this material category and provide insight into how recent formulation modifications may influence adhesive behavior and biological responses.

Nevertheless, several limitations of the present study should be acknowledged. First, the current study evaluated materials under highly controlled laboratory conditions that do not reproduce the complex intraoral environment, including pulpal pressure, dentinal fluid movement, masticatory loading, thermal fluctuations, and long-term hydrolytic degradation. Bond strength was evaluated only after 24 h of water storage, without thermocycling or long-term aging. Adhesive durability under simulated clinical conditions (e.g., thermal and mechanical cycling) is highly relevant for predicting long-term clinical performance. Therefore, the current findings represent initial bond strength rather than long-term stability. Future studies should incorporate thermocycling and prolonged water storage to better assess the durability of the self-adhering flowable composites tested. Second, the EDX analysis was interpreted only qualitatively/semi-quantitatively because of the inherent limitations of standardless EDX analysis and coating-related artifacts. Therefore, no direct relationship between elemental composition and adhesive performance should be overinterpreted. Third, although the present cytotoxicity assays provided valuable information regarding cellular responses, in vitro models cannot fully reproduce the complex oral environment. Extract-based assays, in particular, do not account for the protective role of the remaining dentin barrier or the dilution and clearance of leachables by pulpal blood flow. Additional long-term biological and clinical investigations are necessary to confirm the safety and clinical performance of these materials.

## 5. Conclusions

Within the limitations of this in vitro study, significant differences were observed among the tested self-adhesive flowable composites in terms of dentin bonding performance, interfacial adaptation, mechanical properties, and cytocompatibility. The conventional adhesive system (Clearfil Megabond 2/Clearfil AP-X) exhibited the highest microtensile bond strength and the most favorable resin–dentin interfacial morphology. Among the self-adhesive flowable composites, SA-100R demonstrated superior bonding performance, reduced interfacial gap formation, and higher hardness compared with the other tested materials. In addition, all tested materials exhibited acceptable cytocompatibility, although variations in cell viability and morphology were observed among materials. These findings suggest that recent advances in self-adhesive flowable composite formulations may improve their overall performance; however, their bonding effectiveness remains inferior to that achieved with a conventional adhesive system. Further studies incorporating long-term aging protocols and clinical evaluations are required to confirm the durability and clinical relevance of these findings.

## Figures and Tables

**Table 1:** Adhesives used in this study.

	Materials	Compositions *	Filler Loading *	Application Directions *
Self-adhering flowable composites	SA-100R (Kuraray Noritake Dental, Tokyo, Japan)	Silanated silica filler, hydrophobic aromatic dimethacrylate, ytterbium trifluoride, urethane acrylate, hydrophilic aliphatic methacrylate, silanated colloidal silica, 10-MDP, hydrophilic amide monomer, hydrophobic aliphatic dimethacrylate, 2-dimethylaminoethyl methacrylate, dl-CQ, accelerator, initiators, pigments	N/A	Brush a thin layer (<0.5 mm) onto dentin surface, leave for 10 s and light cure for 20 s. Build additional layers in increments of 2 mm or less and light cure each increment for 20 s.
	Constic (DMG, Hamburg, Germany)	Bis-GMA, EBADMA, UDMA, HEMA, TEGDMA, HDMA, 10-MDP, barium glass filler	43 vol.%	Dispense a thin layer (<0.5 mm) onto dentin surface for 25 s using the brush and light cure for 20 s. Build additional layers in increments of 2 mm or less and light cure each increment for 20 s.
	Fusio Liquid Dentin (Pentron, CA, USA)	UDMA, TEGDMA, HEMA, 4-MET, nano-sized amorphous silica, silane treated barium glass, minor additives, photo curing system	52 vol.%	Dispense a 1 mm increment and agitate to condition the tooth for 20 s prior to light curing for 10 s. Apply additional material in increments of 2 mm and light cure each increment for 10 s.
	Vertise Flow (Kerr Corporation, CA, USA)	GPDM and methacrylateco-monomers pre-polymerized filler, barium glass, nano-sized colloidal silica, nano-sized ytterbium fluoride	44 vol.%	Brush a thin layer (<0.5 mm) with moderate pressure for 15–20 s and light cure for 20 s, Build additional layers (≤2 mm), light cure (20 s)
Two-step self-etch adhesive	Clearfil Megabond 2 (Kuraray Noritake Dental)	Primer: 10-MDP, HEMA, hydrophilic aliphatic dimethacrylate, dl-CQ, water Bond: 10-MDP, Bis-GMA, HEMA, dl-CQ, hydrophobic aliphatic dimethacrylate, initiators, accelerators, silanated colloidal silica	-	Apply the primer and leave for 20 s. Gentle air-blowing for >5 s. Apply the bond. Gentle air-blowing to make the film uniform. Light cure for 10 s.
Conventional resin composite	Clearfil AP-X (APX) (Kuraray Noritake Dental)	Bis-GMA, TEGDMA, silanated barium glass filler, silanated silica filler, silanated colloidal silica, dl-CQ, catalysts, accelerators, pigments	71 vol.%	Apply in layers of maximum 2 mm and light cure for 20 s

* Information as provided by respective manufacturer: bis-GMA: bisphenol-glycidyl methacrylate; EBADMA: ethoxylated bisphenol A dimethacrylate; HDMA: 1,6-hexanediol dimethacrylate; HEMA: hydroxyethyl methacrylate; MDP: 10-methacryloyloxydecyl dihydrogen phosphate; TEGDMA: triethylene glycol dimethacrylate; UDMA: urethane dimethacrylate; 4-META: 4-methacryloxyethyl trimellitic acid; dl-CQ: dl-camphorquinone.

## Data Availability

The data presented in this study are available on request from the corresponding authors.

## Abbreviations

The following abbreviations are used in this manuscript:

SAFCs	Self-adhering flowable composites
µTBS	Microtensile bond strength
KHN	Knoop hardness number
SEM	Scanning electron microscopy
EDX	Energy-dispersive X-ray spectroscopy
hDPSCs	Human dental pulp stem cells
hMSCs	Human mesenchymal stem cells
CCK-8	Cell Counting Kit-8
LIVE/DEAD	Live/dead cell viability assay
ROI	Region of interest
MDP	10-methacryloyloxydecyl dihydrogen phosphate
HEMA	Hydroxyethyl methacrylate
TEGDMA	Triethylene glycol dimethacrylate
Bis-GMA	Bisphenol A-glycidyl methacrylate
UDMA	Urethane dimethacrylate
EBADMA	Ethoxylated bisphenol A dimethacrylate
HDMA	1,6-hexanediol dimethacrylate
4-META	4-methacryloxyethyl trimellitic acid
dl-CQ	dl-camphorquinone
PBS	Phosphate-buffered saline
α-MEM	Alpha minimum essential medium
FBS	Fetal bovine serum
